# Hepatitis C Screening: The Downstream Dissemination of Evolving Guidelines in a Resident Continuity Clinic

**DOI:** 10.7759/cureus.1441

**Published:** 2017-07-07

**Authors:** Kamraan Madhani, Ali Aamar, David Chia

**Affiliations:** 1 Internal Medicine, Yale-New Haven Hospital; 2 Internal Medicine, Yale Waterbury

**Keywords:** hepatitis c virus, preventative health, medical education, quality improvement

## Abstract

Background

In 2012, the Centers for Disease Control and Prevention (CDC) published guidelines supporting one-time screening for hepatitis C (HCV) in all persons born between 1945 and 1965. It is estimated that 75% of adults infected with HCV fall within this cohort. Furthermore, it is projected that this preventative health intervention would lead to the diagnosis of 800,000 unknown cases and the prevention of 120,000 deaths.

Objectives

The primary objectives are to measure adherence to HCV screening in a continuity practice staffed by internal medicine residents and attending physicians and to measure the effect of educational interventions to enhance HCV screening. The secondary objectives include finding whether insurance or provider status affects adherence to HCV screening.

Methods

In 2015, we performed a retrospective chart review of asymptomatic patients born between 1945 and 1965 to estimate the rate of HCV screening. In order to meet inclusion criteria, the patients must have had an HCV status that was unknown and must have been seen by a primary care provider ≥ 2 times between January 1 and December 31, 2013. The data extracted included whether HCV testing was ordered, whether testing was performed primarily for screening purposes, demographic information, insurance status, number of clinic visits, and whether the primary provider was a resident or attending physician. Subsequently, in 2016 we implemented an educational intervention aimed at improving these rates. Afterwards, we repeated the chart review to determine if screening rates had improved.

Results

Out of 294 patients reviewed pre-intervention, 200 patients were eligible for inclusion, of which 17 (8.5%) patients were offered screening for HCV, of which 13 (76.5%) patients completed testing. Following an educational intervention, 484 patients were reviewed and 100 patients were included, of which 34 (34%) patients were screened. Compared to a pre-intervention screening rate of 8.5%, post-intervention screening had improved to 34%, a 300% increase (p<0.001).

Conclusions

Educational interventions are feasible and can lead to significant improvements in clinical practice enabling for the rapid dissemination of evolving guidelines.

## Introduction

Within the United States, there is an estimated 3.2 million people with chronic hepatitis C (HCV) infection, half of whom are unaware of their HCV status [1]. In light of this, the Centers for Disease Control and Prevention (CDC) published guidelines in 2012 supporting one-time screening for HCV in all persons born between 1945 and 1965 given estimates that 75% of adults infected fall within this cohort [1]. Notable at-risk populations who also warrant screening include those with a history of intravenous drug use, hemodialysis, those who received clotting factors before 1987 or blood products before 1992, infection with HIV, and those with persistent elevation in alanine transaminase (ALT) without identifiable etiology. Subsequently, the United States Preventative Services Task Force (USPSTF) released similar recommendations in 2013 [2]. It is projected that this preventative health intervention would lead to the diagnosis of 800,000 previously unknown cases and the prevention of 120,000 deaths.

Prior to the United States Food and Drug Administration (FDA) approval of novel direct-acting anti-viral therapies (DAA), treatment of HCV was arduous and involved interferon-based therapies that carried unfavorable side-effect profiles, which were ultimately intolerable for many patients. Furthermore, treatment with interferon with or without ribavirin was often necessary for 24 or 48 weeks. Even after prolonged combination therapy for 48 weeks, sustained virologic response (SVR), defined as an undetectable serum HCV ribonucleic acid (RNA) level, was reported to be between 38% and 43% of patients with any HCV genotype [3-4]. However, the landscape of HCV treatment changed dramatically as these new, less toxic DAA medications have become available, demonstrating consistent ability to achieve SVR of ≥ 93% of patients with genotype 1 who were treated for as little as eight weeks [5]. Moreover, studies following patients treated for 12 or 24 weeks have reported SVR of up to 99% in patietns with genotype 1 [5-6]. Given how treatable HCV has become, it is even more imperative to ensure those infected are identified promptly to treat them before they develop the long-term complications of HCV, namely cirrhosis and premature death.

The downstream dissemination of the CDC and USPSTF recommendations regarding HCV screening remains challenging. Obstacles that are easily overcome include educating physicians on the prevalence of HCV, the ease of testing, and the advancements in treatment options. Unfortunately, even equipped with this information, the lack of time during short office visits often results in incomplete or postponed health care maintenance. This is especially true in resident-run community health centers where there is a constant flux of housestaff between the inpatient and outpatient settings. Through this project, we primarily aimed to measure and improve screening practices in a continuity clinic staffed by internal medicine residents through the dissemination of current screening guidelines and to determine whether provider status or insurance coverage influences HCV screening adherence.

## Materials and methods

In 2015, we performed a retrospective electronic chart review of a random sample of patients born between 1945 and 1965 who received their primary care at an academic continuity practice staffed by internal medicine residents and attending physicians (Waterbury, Connecticut). In order to meet inclusion criteria for the study, patients must have been born between 1945 and 1965, had an HCV status that was unknown, and had been seen by a primary care provider ≥2 times between January 1 and December 31, 2013. Patients were included based on a minimum of two visits with a primary provider within the clinic to allow for sufficient opportunity for providers to discuss and offer appropriate screening. Patients with HCV testing for any purpose other than routine screening were excluded, e.g. for evaluation of elevated transaminase levels in the assessment of abdominal pain or workup of new onset jaundice. The data extracted included demographic information, known HCV risk factors [1], insurance status, number of clinic visits, whether the primary provider was a resident or attending physician, whether HCV screening was offered to the patient, whether the patient accepted or declined to be screened, whether HCV antibody testing was ordered and subsequently completed by the patient.

In 2016, after initial data was extracted, a multifactorial educational intervention was implemented. The intervention included: (1) brief pre-clinic conferences focused on current HCV screening guidelines delivered every two weeks coinciding with resident rotation start dates; (2) email reminders outlining screening recommendations to physician providers sent at two-week intervals coinciding with rotation start dates; (3) written reminders in physician work areas; (4) informational posters in examination rooms explaining HCV testing and indications to prompt patient inquiry regarding screening. The educational intervention was enacted for a four-month period between January 1 and April 30, 2016. Subsequently, a post-intervention electronic chart review was performed and included patients seen by a primary care provider ≥ 2 times in the following two months between May 1 and June 30, 2016, in order to measure the effectiveness of the intervention.

Categorical variables (gender, insurance status, provider status) were compared using chi-squared testing, while continuous variables (age, number of visits) were compared using the t-test. A p-value of <0.05 was considered significant. IRB approval was sought and obtained prior to the initiation of the study.

## Results

**Figure 1:**
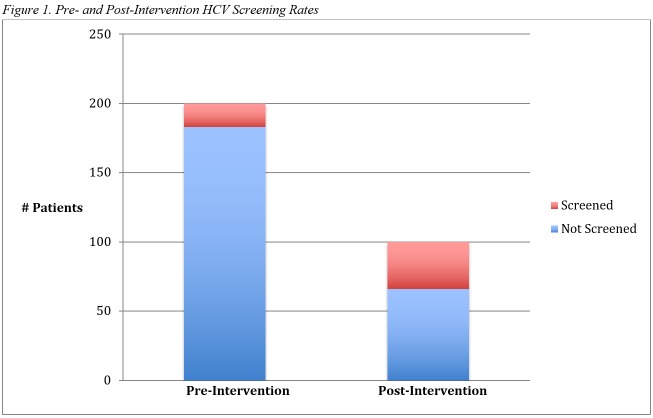
Pre- and Post-Intervention HCV Screening Rates

Pre-intervention, a total of 294 patients were randomly reviewed, and a total of 200 patients met the criteria for inclusion. Eighty-eight (44%) patients were insured by Medicaid, 77 (38.5%) patients by Medicare, 32 (16%) patients with private insurance, and three (1.5%) patients were self-pay. One hundred and twenty-one (121) (60.5%) patients had resident physicians, and 79 (39.5%) patients had attending physicians as their primary care providers. Only 17 (8.5%) patients were offered screening for HCV. All 17 patients accepted screening, of which 13 (76.5%) patients completed the test, and none tested positive. There were no statistically significant differences in HCV screening rates when comparing insurance status (p=0.96), provider status (p=0.72) or number of clinic visits (p=0.15) (Tables 1-2, Figure 1).

**Table 1:** Pre-Intervention Results for HCV Screening

Demographics	Total N (%)	Screening Not Offered N (%)	Screening Offered N (%)	p-value
Gender				0.71
Male	72 (36)	66 (36.1)	6 (35.3)	
Female	128 (64)	117 (63.9)	11 (64.7)	
Age (Mean ± SD)	60 ± 5.8	60 ± 5.7	61 ± 7.1	0.58
# Visits (Mean ± SD)	5.5 ± 3.1	5.4 ± 3.2	6.5 ± 2.3	0.15
Provider Status				0.72
Resident	120 (60.6)	109 (60.2)	11 (64.7)	
Attending	78 (39.4)	72 (39.8)	6 (35.3)	
Insurance Provider				0.96
Medicare	77 (38.5)	73 (94.8)	5 (5.2)	
Medicaid	86 (43)	76 (88.4)	10 (11.6)	

**Table 2:** Post-Intervention Results for HCV Screening

Demographics	Total N (%)	Screening Not Offered N (%)	Screening Offered N (%)	p-value
Gender				0.37
Male	38 (38)	23 (34.8)	15 (44.1)	
Female	62 (62)	43 (65.2)	19 (55.9)	
Age (Mean ± SD)	58 ± 5.0	58 ± 4.8	58 ± 5.6	0.81
# Visits (Mean ± SD)	3.1 ± 1.0	3.1 ± 1.3	3.3 ± 1.5	0.39

Post-intervention, a total of 484 patients were randomly reviewed, and a total of 100 patients met the criteria for inclusion. Thirty-four (34%) patients were offered screening for HCV appropriately, of which 32 (94.1%) patients accepted screening, 13 (38.2%) completed the test HCV screening, and one patient was newly diagnosed with HCV and referred for treatment. Comparing pre- and post-intervention results, HCV screening rates by providers increased from 8.5% to 34% (p<0.001), a percent increase of 300% in screening practices. However, completion of HCV testing was higher in the pre-intervention cohort for unclear reasons (Table 1, Figure 1).

## Discussion

Adherence to HCV screening guidelines in our resident continuity practice was low. This is consistent with screening rates of 4.3% reported by other centers [7], thus highlighting that the dissemination of evolving guidelines requires practice-based improvement and vigilance of the ever-changing landscape of evidence-based medicine.

It is estimated that approximately 50% of persons infected with HCV are unaware that they are infected and the largest sub-group of persons affected are those born between 1945 and 1965 [1]. Regular e-mail and written reminders, educational conferences, and informational posters are all low cost and easily implementable strategies that raise awareness and improve screening practices. Adding automatically triggered reminders for eligible patients through an electronic medical record could increase screening rates even further [8-9].

This simple intervention can help to decrease the current burden of disease as well as prevent significant morbidity and mortality associated with untreated infection including cirrhosis, end stage liver disease, and hepatocellular carcinoma (HCC). Furthermore, by preventing progression to end stage liver disease, HCV-related deaths, which are projected to peak between 2030 and 2035, can be substantially decreased [10-11].

Limitations of our study include the retrospective nature and dependence on thorough and clear documentation by providers. Without documentation of whether a discussion was conducted regarding HCV screening, we would have no way of tracking whether HCV screening was offered to patients in our study. While our study looked exclusively at patients born between 1945 and 1965, other indications for HCV screening (such as intravenous drug use), was beyond the scope of this study.

## Conclusions

In conclusion, the consequence of missing any of the estimated 800,000 unknown cases of HCV in the United States can be catastrophic. If left undiagnosed, HCV can lead to cirrhosis, hepatocellular carcinoma and other decompensated conditions with severe morbidity and mortality. The ownership thus falls to primary care physicians to be diligent in adherence to important screening guidelines. Educational interventions may assist in augmenting our capacity to prevent the aforementioned complications of chronic HCV infection through early detection and treatment.
